# Cost Saving Opportunities in NSCLC Therapy by Optimized Diagnostics

**DOI:** 10.3390/cancers9070088

**Published:** 2017-07-11

**Authors:** Ilija Nenadić, Janine Staber, Susanne Dreier, Guus Simons, Verena Schildgen, Michael Brockmann, Oliver Schildgen

**Affiliations:** 1 Kliniken der Stadt Köln gGmbH, Klinikum der Privaten Universität Witten/Herdecke mit Sitz in Köln, Institut für Pathologie, D-51109 Cologne, Germany; nenadici@kliniken-koeln.de (I.N.); schildgenv@kliniken-koeln.de (V.S.); brockmannm@kliniken-koeln.de (M.B.); 2 APOLLON Hochschule der Gesundheitswirtschaft, D-28359 Bremen, Germany; janinestaber@gesundheit.bremen.de (J.S.); susanne.dreier@bfh.ch (S.D.); 3 PathoFinder, 6229 Maastricht, The Netherlands; guus.simons@pathofinder.com

**Keywords:** ALK, crizotinib, cost saving, lung cancer, NSCLC

## Abstract

With an incidence of 68 new cases per 100,000 people per year, an estimated total number of up to 350,000 new non-small-cell lung cancer (NSCLC) cases are diagnosed each year in the European Union. Up to 10% of NSCLC patients are eligible for therapy with novel ALK (anaplastic lymphoma kinase) inhibitors, as they have been diagnosed with a mutation in the gene coding for ALK. The ALK inhibitor therapy costs add up to approx. 9000 € per patient per month, with treatment durations of up to one year. Recent studies have shown that up to 10% of ALK cases are misdiagnosed by nearly 40% of pathologic investigations. The current state-of-the-art ALK diagnostic procedure comprises a Fluorescent in situ Hybridization (FISH) assay accompanied by ALK inhibitor therapy (Crizotinib). The therapy success ranges between a full therapy failure and the complete remission of the tumor (i.e., healing), but the biomedical and systemic reasons for this range remain unknown so far. It appears that the variety of different ALK mutations and variants contributes to the discrepancy in therapy results. Although the major known fusion partner for ALK in NSCLC is the Echinoderm microtubule-associated protein-like 4 (EML4), of which a minimum of 15 variants have been described, an additional 20 further ALK fusion variants with other genes are known, of which three have already been found in NSCLC. We hypothesize that the wide variety of known (and unknown) ALK mutations is associated with a variable therapy success, thus rendering current companion diagnostic procedures (FISH) and therapy (Crizotinib) only partly applicable in ALK-related NSCLC treatment. In cell culture, differing sensitivity to Crizotinib has been shown for some fusion variants, but it is as yet unknown which of them are really biologically active in cancer patients, and how the respective variants affect the response to Crizotinib treatment. Moreover, it has been demonstrated that translocated ALK genes can also be observed in healthy tissues and are not compulsorily associated with tumors. Therefore, it is important to keep in mind that even for the known variants of ALK fusion genes, the biological function is not known for all variants, and that no information is available on the homogeneity of ALK fusion variants within a single tumor. These facts, in concert with data for ALK mutation prevalence and therapy outcomes of a German cohort of NSCLC patients, support the hypothesis that, by using novel companion diagnostic tools in combination with therapy outcome predictions, massive cost savings could be possible in European Health Care systems without a loss of patient care.

## 1. Introduction

In the era of personalized medicine, the molecular diagnostics of non-small cell lung cancer (NSCLC) have become more and more complex, and therapeutic interventions nowadays are highly targeted, and sometimes restricted to narrow clinical entities [1,2,3,4,5,6]. Lung carcinomas, in contrast to other carcinomas, are characterized by a relatively high frequency of genetic alterations [7].

An example for such a narrow therapy window is the tyrosine kinase inhibitor (TKI) treatment of ALK-mutated NSCLC. According to several studies, the prevalence of ALK-positive NSCLC ranges between 2% and 7% of the entire NSCLC cohort [7,8,9,10,11,12]. ALK mutations are usually detected by either a FISH assay or by immunohistochemistry, and for both methods CE IVD- and even FDA-approved assays exist [12,13].

The average cost for ALK-specific TKI ranges between 6000 and 9000 €, while the costs for ALK diagnostics vary between 40 and 80 € per assay [14,15]. In addition, it is important to note that the median increase in progression free survival under ALK-specific TKI therapy is 4 months [16,17,18,19], with a range between 2 months and total remission [16,17,18,19]. Taking into account that ALK fusion mutations can theoretically occur with up to 21 fusion partner genes, of which even EML4 can form up to 18 variants, this latter fact implies that an optimized diagnostic algorithm accompanied by a prediction software tool that correlates the likelihood of therapy response of a variant (as determined in vitro) is capable of saving enormous therapy costs by excluding those variants that are not susceptible to TKI therapy. Based on these considerations, we addressed the question of how much the development of such an assay would cost, how much the assay itself would cost, and how much could be saved in healthcare reimbursement budgets by such an assay. As a basis, we have extrapolated data from our own clinical cohort of NSCLC patients with FISH-confirmed ALK mutation and epidemiological data reported to and published by the Robert-Koch-Institute.

## 2. Results

Lung cancer is one of the most frequent cancers worldwide. According to the Robert Koch Institute (RKI) data on NSCLC in Germany, approximately 35,000 male and 20,300 female patients are newly diagnosed with lung cancer per year, with a low five year survival rate of 21% for female and 16% for male patients in 2012 [20].

Approximately 40–50% of patients with NSCLC are tested in stage IV, while NSCLC includes 85% of all newly diagnosed lung cancers [21]. Taking into account the data from the RKI and the prognosis for 2016, 85% of 55,300 patients, i.e., 47,005 patients, are likely to be diagnosed with NSCLC each year. Of those patients, 18,802–23,503 will be diagnosed in stage IV (i.e., 40–50%). Approximately half to one third of those patients will be eligible for ALK testing.

As shown recently, ALK testing methods require significant improvement: von Laffert and colleagues have shown that, in up to 10 percent of clinical cases, ALK diagnostics were incorrect in up to 40% of pathology departments, although in most cases FDA-approved methods were used [22,23]. This diagnostic challenge is complicated by the fact that there is an increasing number of identified ALK variants in NSCLC for which the prevalence, the treatment response, and clinical significance remain unknown [1,2]. Whilst the overall positivity rate of mutated ALK in NSCLC is published as being between 2 and 7 percent, the response rate to the ALK inhibitor Crizotinib was described as 57%, with a remarkably high rate of progression-free survival of 72% after 6 months of ALK-inhibitor therapy [17,24]. Thereby, the response rate ranges between full remission and total lack of response, a phenomenon that can most likely be attributed to the high number of different variants. Unfortunately, this topic has not yet been addressed, and only the median progression-free survival during ALK-inhibitor therapy has been published, with an increased rate of 7.7 vs. 3 months, i.e., a median improvement of 4 months [25]. Therefore, it was our aim to analyze to what extent these internationally accepted data also apply to the situation in Germany in general and to the clinical cohort in our hospital in particular.

Our cohort consisted of patients in Cologne, and the region within approximately 100 km of Cologne (Table 1). The patients coming to our hospital were either transferred to our hospital because of a pre-diagnosed NSCLC or because of clinical symptoms of lung cancers. Therefore, no limitations existed with respect to age, sex, or ethnic background; thus, the cohort is not a “study-cohort”, but reflects daily hospital practice. In our cohort in 2014, 645 patients were diagnosed with lung cancer, of which 574 suffered from NSCLC. The total number of NSCLC during the entire observation period was 1722 registered patients with NSCLC. Of those, 860 patients were in NSCLC stage IV. A total number of 60 patients out of the 860 NSCLC stage IV patients tested positive for ALK mutation, and thus in principle were eligible for Crizotinib therapy. This latter number corresponds to a percentage of 3.8% ALK-positive NSCLC in the entire cohort, thus being in a range comparable to other cohorts [26]. Out of the 60 patients who were, in principle, eligible for Crizotinib therapy, only 22 received Crizotinib therapy, whilst the remaining patients received alternative or palliative therapy.

Out of the 22 patients receiving Crizotinib therapy, 14 were male (63.6%) and 8 were female (36.4%). The mean age was 62.68 years, ranging between 48 and 79 years, with a median age of 62.5 years. Seven patients were smokers, 11 patients were former smokers, 3 patients had never been smokers, and for one patient the smoking status remained unknown. The therapy duration had a mean of 5.36 months, whilst the median therapy duration was 2 months, and thus significantly lower than in other published studies. The majority of patients had therapy durations of max. 4 months (16 out of 22, i.e., 72.7%); only 13.6% were still receiving therapy at the cut-off date of this study. Serious differences were also observed in the clinical courses during treatment. Three patients died within the month when the Crizotinib therapy was initiated, while another patient died 28 months after the therapy start. In one case (patient 1), the therapy had to be stopped due to side effects, and in one case the daily dose was reduced after two weeks. Six patients received an additional therapy (chemotherapy or radiation therapy). Only one patient had a stable remission, and continued to receive Crizotinib therapy even 25 months after therapy onset.

In the case of the cohort described above, the cost for the ALK-inhibitor therapy was 118 months × 6087.77 = 718,356.86 € (Table 2). No further differences in therapy costs could be identified between ALK-positive and ALK-negative patients. It was surprising that in our patient cohort the median and the average therapy duration were below those of published studies. Thereby, it has to be mentioned that our cohort was not a “controlled” study cohort but an observational study, and reflected the “real life” daily patients coming to a German hospital specialized in the therapy of lung diseases.

The costs for ALK testing with immunohistochemistry (IHC) or fluorescence in situ hybridization range between 80 and 90 € per sample. This calculation is based on the list prices for ALK FISH assays from the German branches of vendors of ALK FISH assays (Abbott, Wiesbaden, Germany; ZytoVision, Bremerhafen, Germany; KreaTech, Amsterdam, The Netherlands). The cost for the novel assay was calculated by analogy to the costs of Respifinder and Meningofinder assays (multiplex assays making use of the same technology proposed here) by our co-author Guus Simons (CEO of Pathofinder, Maastricht, Germany). We calculated that a novel assay that can distinguish between all known ALK fusion variants, including other variants that determine eligibility for ALK-inhibitor therapies like ROS fusions, would also cost about 80 € per sample. Such an assay could be based on MLPA and preamplification as already used for other purposes by PathoFinder, and could include a software tool that enables therapy outcome predictions based on cell culture and correlated therapy data. Therefore, the initial development costs for such an assay would reach a maximum of 6 million € as a single investment (our exact calculation, based on a Horizon2020 grant proposal submitted to the European Commission, was 5,183,566 €). The proposed assay should undergo clinical evaluation; that is, the clinical outcome of therapy in relation to ALK fusion variants needs to be included in the interpretation software tool. This goal can be easily achieved by combining cell culture data with cell culture phenotyping assays, making use of the different variants and clinical observations. The in vitro phenotyping would either be based on cell lines with known ALK mutations, or these mutations would have to be included in the cells by recombination technology like CRISPR/Cas or Cre/loxP assays. The predictions should be based on the combinations of in vitro and in vivo data. If a variant is not treatable in vitro, it won’t be treatable in vivo. Thereby, dose effects and toxicity profiles have to be taken into account. The prediction software should be constructed by analogy with the Geno2pheno software used for HIV therapy outcome prediction, and make use of machine learning approaches.

Based on these latter specifications, we then assumed that such an assay could have predicted the outcome of Crizotinib therapy in our patient cohort, i.e., the 6 out of 22 patients (27.3%) treated could have been predicted as responders, whilst the remaining patients could have been identified as non-responders (72.7%); thus, the high-cost Crizotinib therapy could have been avoided in 72.7% of cases, resulting in a remarkable cost-saving potential (Table 2).

The frequency of ALK translocations in NSCLC was published as 1–2% [27]; i.e., based on the above-mentioned epidemiological data of approximately 47,005 newly diagnosed NSCLC patients [28] per year, a minimum of between 470 and 940 patients per year would be eligible for ALK testing in Germany.

Taking international studies into account, the frequency of ALK translocations would be 4–6%, resulting in a maximum of 1880–2820 patients per year being eligible for ALK-inhibitor therapy.

Excluding 2 patients of our cohort of 22 ALK-positive patients, where in one case Crizotinib therapy was stopped and in the other, therapy was initiated at the cut-off point of this study, the remaining 20 ALK-positive patients were used for the subsequent calculations. Of these 20 ALK-positive patients, we calculated a number of 7.33 ALK-positive patients under Crizotinib therapy; i.e., our cohort reflects 20/940 = 2.13% to 20/470 = 4.26% of German patients with ALK therapy, i.e., (470/20) × 7.33 = 172 to (940/20) × 7.33 = 355 patients per year in Germany under Crizotinib therapy in Germany (data from Table 2).

Based on these calculations, the average yearly therapy duration is 172 to 355 patients × 5.36 months = 921.92 to 1902.8 months, with a median yearly therapy duration of 172 to 355 × 2 months = 344 to 710 months.

Based on a cost of 6087.77 € per month for Crizotinib therapy [29], the average costs for Crizotinib therapy in Germany could be estimated by 921.92 to 1902.8 months × 6087.77 € = 5,612,436.92 to 11,583,808.76 €, with median therapy costs of 344 to 710 months × 6087.77 € = 2,094,192.88 to 4,322,316.70 €. As the international published data for the prevalence of mutated ALK are 4–6% (see above), these costs have to be multiplied by a factor of 2, resulting in a maximum average cost for Crizotinib therapy of 23,167,617.52 €, and a median cost of 8,644,633.40 €, respectively [30].

Using the data from Table 1 and Table 2 as a basis, the cost-saving potential for our patient cohort would have been 31 months × 6087.77 € = 188,720.87 €, if the non-responder rate of 72.73% had been predicted in advance of therapy. Extrapolated to the estimation for the entire German cohort, the cost-saving potential would be 4,081,772.31 €–8,424,588.15 € if the average therapy duration is used as the basis for the calculation, and 1,523,049.37 €–3,143,503.05 € if the median therapy duration is used as the basis for the calculation.

Taking into account the overall population of the European Union, which is estimated to be 510.1 million people [31], the putative cost-saving potential would be 11,006,270.64 €–22,012,541.27 € per year based on the average therapy duration of non-responders, and 11,361,311.57 €–22,722,623.14 € based on the median therapy duration of non-responders, per year (Table 3).

## 3. Discussion

The currently published data for ALK-inhibitor therapy in concert support the hypothesis that the failure of ALK therapy is most likely caused by distinct variants of ALK fusion genes. It is therefore essential to develop diagnostic tools that can discriminate between those variants and would be able to predict the therapy outcome.

This goal can be achieved: We propose the optimization of diagnosis and therapy of lung cancer subgroups in Europe. This must include the collection of biological research data (ALK fusions, ROS-/RET-fusions) from a European patient cohort, using a novel technology for 5′ fusion partner identification. Additionally, patients’ retrospective medical data (effectiveness of treatment related to distinct ALK variants) have to be collected in the same database. Moreover, it is crucial to start the characterization of ALK-positive lung cancer by developing computational statistical model/machine learning approaches in which all biological and medical data are correlated in order to fully understand the biological interactions of ALK fusions and beyond ROS/RET fusions. Finally, it is possible to start the development of a multiplex PCR detection assay for the detection of ALK fusion partners in NSCLC patients based on the databases mentioned above, and to enable automated diagnosis of ALK fusion partners that predicts personalized treatment or outcomes of treatment.

This goal may appear difficult, as the treatment of ALK patients appears complex, but experiences in other disciplines of personalized medicine have repeatedly proven this concept. The most prominent example is the prediction of antiretroviral therapy outcomes in advance of therapy based solely on the genotypic information [32,33,34,35,36,37]. In this so-called geno-to-pheno prediction, HIV resistances and the most likely therapy outcomes can be predicted, even though the number of HIV-genotype variants and the available drugs exceed the situation in ALK-inhibitor therapy. Consequently, we recommend initiating public initiatives to improve ALK diagnostics in NSCLC, to optimize the subsequent therapy, and to focus on the best available treatment options, even if it may not be a newly available drug.

## 4. Materials and Methods

As shown in previous studies, our cohort of NSCLC patients reflects a typical European cohort of this clinical entity [38,39,40,41]. We analyzed how many patients of our cohort were susceptible for Crizotinib therapy based on FISH- and IHC-positive ALK-mutated NSCLC, and followed up the therapy of those patients. These analyses were in accordance with a vote from the local ethical committee (Ethical Committee of the Private University of Witten/Herdecke, vote no. 86/2014, Witten, Germany). We therefore made a cohort analysis of our own patients, treated in our hospital between 2012 and 2015. The cohort included primary therapy data from 60 patients with advanced NSCLC and ALK translocation.

The economic analysis includes a micro-analysis for our hospital and a macro-analysis upscale (see below). We chose the perspective of health insurance providers, and focused thereby solely on economic aspects, without taking into account non-monetary effects such as life quality aspects (e.g., no side effects by false positive treatments or placebo effects in cases where Crizotinib is unlikely to be efficient).

We then calculated the costs for Crizotinib therapy based on published data and list prices of the drug per month. Taking into account the epidemiological data available for NSCLC in Germany and the population of the European community we extrapolated these calculations in the form of a min/max cost analysis, and analyzed the putative annual cost savings for NSCLC therapy in Europe.

Our study presumes the hypothesis that it is possible to develop a novel diagnostic assay based on the PathoFinder technology that is able to determine the ALK fusion variant and predict the therapeutic outcome of ALK-inhibitor therapy based on a machine-learning database built on experimental and epidemiological data collected in a multicenter study, and is available at a cost of 80–90 € per sample, with a single assay development investment of 3–6 million € [42,43].

## 5. Conclusions

In this study, we proposed a way to reduce therapy costs and medical side effects in NSCLC therapy by improving molecular diagnostics. We exclusively focused on a single multiplex assay, but have to acknowledge that other assays, like next-generation sequencing, may result in even greater economic effects than our putative assay would do. Moreover, one of the reviewers of the manuscript suggested the idea of extending our approach to further markers, and we agree with this reviewer’s conclusion that the final goal should be a comprehensive tumor gen2pheno tool that predicts the likely outcome of a therapy option in a given tumor-mutation profile, thus guiding therapy and avoiding costs, while delivering the best available therapy option for the individual patient.

## Figures and Tables

**Table 1:** Overview on the patient cohort that was used as the basis for the subsequent health-economic analyses (target date for ongoing therapy 30 June 2016).

Patient No.	Age at Therapy Start (Years)	Sex	Smoking Status	Length of Crizotinib Therapy (Month)	Exitus Letalis Post Therapy (Month)
1	75	female	ex-smoker	1	
2	69	female	never-smoker	15	28
3	75	male	ex-smoker	2	2
4	50	female	ex-smoker	15	15
5	74	male	ex-smoker	4	
6	62	male	smoker	9	
7	73	male	smoker	1	1
8	76	female	ex-smoker	25, ongoing	
9	65	male	ex-smoker	2	
10	48	male	ex-smoker	1	
11	63	male	smoker	2	3
12	56	male	ex-smoker	1	1
13	53	female	smoker	4	
14	55	male	never-smoker	1, ongoing	
15	48	female	ex-smoker	1	2
16	62	female	ex-smoker	2	
17	65	female	smoker	2	
18	71	male	ex-smoker	2	2
19	54	male	unknown	15	
20	51	male	never-smoker	4	
21	55	male	smoker	1	1
22	79	male	ex-smoker	8, ongoing	
	mean: 62.68 years			average therapy duration: 5.36 months	
	median: 62.5 years			median therapy duration: 2 months	

**Table 2:** Cost calculation for ALK-inhibitor therapy in our hospital and extrapolation of costs for Germany per year. * (data for 2015 and 2016 not fully available); ** (extrapolation based on the portion of patients under ALK-inhibitor therapy in our hospital); *** (These costs are a rough estimation as the costs for the drugs may vary between EU member states).

Clinical and Therapy Information	Our Hospital	Germany	European Union
patients with lung carcinoma	645 (in 2014) *	55,300 (2016)	411,765 (extrapolated)
patients with NSCLC	574 (in 2014) *	47,005 (85%)	350,000 (85%)
number of ALK positive patients eligible for ALK therapy ≥1 month	20 (per year from 2012–2015)	470–940 (1%–2% of NSCLC)	3500–7000 (1%–2% of NSCLC)
number of ALK positive patients treated per year	7.33	172–355 **	1283–2566 **
average duration of ALK-inhibitor therapy	5.36 months		
median duration of ALK-inhibitor therapy	2 months		
total cumulated average therapy duration per year	39.29 months	921.92 months–1902.8 months **	6877.1 months–13,754.2 months **
total cumulated median therapy duration per year	14.66 months	344–710 months **	2566–5132 months **
total costs per year based on average therapy duration	239,181.18 €	5,612,436.92 €–11,583,808.76 € **	41,864,864.11 €–83,729,728.22 € ***
total costs per year based on median therapy duration	89,246.71 €	2,094,192.88 €–4,322,316.70 € **	15,621,218.14 €–31,242,436.27 € **

**Table 3:** Calculation of the yearly cost-saving potential.

	Kliniken der Stadt Köln gGmbH	Germany	European Union
number of ALK-positive patients with ALK-inhibitor therapy per year	7.33	172–355	1283–2566
non-responders	72.73%		
cost-saving potential based on average therapy duration of non-responders	62,880.72 €	1,475,509.41 €–3,045,382.78 €	11,006,270.64 €–22,012,541.27 €
cost-saving potential based on median therapy duration of non-responders	64,909.13 €	1,523,106.48 €–3,143,620.94 €	11,361,311.57 €–22,722,623.14 €
average cost-saving potential calculated on our cohort	219,096.56 €	5,141,147.77 €	38,349,370.60 €
median cost-saving potential calculated on our cohort	226,164.22 €	5,306,991.22 €	39,586,452.15 €
